# Environmental Investigation and Surveillance for *Legionella* in Aotearoa New Zealand, 2000–2020

**DOI:** 10.1007/s00284-023-03261-9

**Published:** 2023-03-30

**Authors:** Frances F. Graham, David J. G. Harte, Michael G. Baker

**Affiliations:** 1 Department of Public Health, University of Otago, P. O. 7343, Wellington South, 6242 New Zealand; 2 ESR, Legionella Reference Laboratory, Health Programme, Kenepuru Science Centre, Wellington, New Zealand

## Abstract

The reported rate of legionellosis is increasing in Aotearoa New Zealand (NZ) with most cases community-acquired, sporadic (non-outbreak) and without an identifiable source. This analysis used two datasets to describe the environmental sources that contribute to *Legionella* in NZ, based on linkages with outbreaks and sporadic clinical cases, and analysis of environmental testing data. These findings highlight the need for enhanced environmental investigation of clinical cases and outbreaks. There is also a need for systematic surveillance testing of high-risk source environments to support more rigorous controls to prevent legionellosis.

**Supplementary Information:**

The online version contains supplementary material available at 10.1007/s00284-023-03261-9.

## Introduction

Legionellosis is caused by the gram-negative bacterium *Legionella*. This infection is the consequence of environmental exposure to pathogenic legionellae that are ubiquitous in both water and moist soil ecosystems. The severity of the disease varies from a mild febrile illness (Pontiac fever) (incubation period commonly 24–48 h) [1] to a serious and sometimes fatal form of pneumonia (Legionnaires’ disease) (incubation period commonly 2–10 days) [2]. In Aotearoa New Zealand (NZ), legionellosis has been a notifiable disease since June 1980 [3]. Inhalation of aerosolized bacteria from an environmental source is the usual means of *Legionella* transmission, usually from engineered environments such as wet cooling towers and water distribution systems, which act as reservoirs and amplifiers of the bacteria [1]. Furthermore, the inhalation of contaminated aerosolized dust from the handling of compost and potting mix is likely to be an important transmission route that contributes to the cases of legionellosis caused by *Legionella longbeachae* [4], which is the predominant species causing disease in NZ [5].

In NZ sporadic community cases of legionellosis comprise at least 90% of all reported cases compared to travel-associated (6.5%, [3]) and hospital-acquired (< 1%, [6]). A total of 2628 cases of legionellosis were notified between 2000 and 2020 (representing an overall mean annual incidence rate of 2.7/100 000 population). The mean annual incidence rate increased from 1.6/100 000 population in 2000–2009 to 3.9/100 000 population in 2010–2020, with much of this increase attributed to improved awareness and testing [6].

NZ has limited public health resources, which means that there is likely to be variability in the intensity of case investigations nationally, that is, whether sampling is deemed appropriate as part of a case follow up. Public health practitioners need to know the sources of sporadic *Legionella* so they can effectively target control measures [7].

The goal of this study is to provide a finer lens on the environmental sources of *Legionella* that contribute to human disease in NZ to support improved prevention and control measures. Specific aims are to: (1) describe the environmental sources identified for outbreaks of legionellosis; (2) describe the sources implicated for sporadic cases; and (3) summarize the extensive environmental sampling data collected from a variety of sources. These aims seek to address a knowledge gap identified in the literature on the lack of *Legionella* studies that support definite and probable ranked sources [7].

## Methods

In NZ legionellosis surveillance (including cases of LD and Pontiac fever) comprises both laboratory and disease notification data (including outbreaks) collected for public health purposes [3]. Surveillance data is collated by the Institute of Environmental Science and Research (ESR) on behalf of the NZ Ministry of Health. Outbreaks are defined as two or more cases associated with a single identified site of exposure with dates of onset within 6 months of each other [8]. Data on legionellosis outbreaks reported from 2000–2020 were obtained from ESR. The outbreaks were delineated by monthly distribution, location, outbreak source, *Legionella* species (spp.) and serogroup (sg), number of confirmed cases, hospitalizations, and deaths.

We analysed source data for *Legionella* bacteria isolated from all environmental sources collected by the ESR Legionella Laboratory between 2000 and 2020. These included all of those identified from case and outbreak investigations and from systematic environmental testing conducted for risk assessment and compliance testing purposes. The data (all environmental isolates) were amalgamated to assess the prevalence of different *Legionella* strains from the full range of source categories (known reservoirs and exposure sources for this bacteria). A further analysis of *Legionella* culture isolates by infective organism (species and serogroup) from environmental source category for the 5-year period 2016–2020 was also undertaken. For the purpose of this study, we have used a classification system adapted from Orkis et al., 2018 [7] with three defined categories: a definitive source where there is a molecular match (using sequence-based typing [9] or *mip* gene sequence analysis [10] between the clinical isolate and the environmental isolate from the source to which the case was exposed during their incubation period; a probable source where there is a species match between the case and their environmental source but identification was made using incongruent methods, such as serology for the clinical case and culture for the linked environmental source; and a suspected source where there is a likely source identified, but no environmental sampling is undertaken; for example, cases of *L. longbeachae* infection where the interviewed case reports using compost during the incubation period, no other exposure risks were identified, but no sampling/testing of the compost materials was undertaken. We also include data for the clinical cases where no infection source was indicated, and no environmental sampling was undertaken.

## Results and Discussion

### *Legionella* Outbreaks

Seventeen outbreaks with an identified source were detected during the 2000–2020 period (Table 1). These were mainly traced to either a cooling tower, a spa pool, or a compost source. No outbreaks were identified in years 2001, 2004, 2008–2010, 2012, 2014, and 2016–2019, indicating that these events are not commonly identified in NZ. In most years when outbreaks were reported, there were multiple outbreaks.

**Table 1:** Recorded outbreaks of legionellosis, and their identified sources, New Zealand, by year, 2000–2020

Year	Month	Location	Identified source	Species & serogroup	Concentration at source	No. of Legionella cases			Number hospitalized	No. of deaths
						Total	Confirmed	Probable		
2020	Nov–Dec	Paihia	Public spa pool	Lpsg1	88 cfu/mL	3	3		2	
2020	Sep	Christchurch	Compost	Llsg1	ND	2	2		1	
2015	Aug–Nov	Pahiatua	Cooling tower	Lpsg1	10–640 cfu/mL	13	3	1	3	
				Llsg1	170–180 cfu/mL		1	4		
				Llsg2	ND			1		
				Lsth	ND			2		
				Lpsg5	ND			1		
2015	Apr	Christchurch	Cooling tower	Lpsg1	420 cfu/mL	6	6		6	
2013	Mar–Sep	Hokitika	Public Spa pool	Lpsg1	47 cfu/mL	2	2		2	
2011	Apr	Blenheim	Cooling tower	Lpsg1	1400 cfu/mL	14	6	8	3	
2007	Jan	Gisborne	Potting mix	Llsg2	ND	9	9		3	
2006	Feb-Mar	Beachlands	Rainwater tanks	Lpsg1	80–800 (ave 340) cfu/mL	4	3	1	1	1
2005	Aug	North Shore	Domestic spa pool	Lpsg1	100 cfu/mL	2	2			
2005	Apr-Aug	Christchurch	Cooling tower	Lpsg1	2400 cfu/mL	19	19		19	3
2003	Dec	South Auckland	Compost	Llsg1	ND	2	2		2	
2003	Nov	North Shore	Compost	Llsg1	ND	2	2		1	
2003	Oct-Nov	Upper Hutt	Composting facility	Llsg1	ND	4	3	1	3	
2003	Oct	Wellington	Display spa pool	Lpsg2	ND	3	3		3	
2002	Dec	Auckland	Display spa pool	Lpsg2	> 500 cfu/mL	3	3		3	1
2000	Oct-Nov	Auckland	Cooling tower	Ldum	600 cfu/mL	2	2			
2000	Sep	Whangarei	Compost	Llsg1	ND	2	2		2	

Ldum = *L. dumoffii*; Ll = *Legionella longbeachae*; Lp = *Legionella pneumophila*; Lsth = *L. sainthelensi*; sg = serogroup; ND: Not determined; cfu = colony forming unit

The 92 outbreak cases represented 3.5% of all notifications (*N* = 2628) for this period. The number of legionellosis cases associated with these outbreaks ranged from two to 19. A total of 54 outbreak-associated cases were hospitalized (hospitalization information was not recorded for one outbreak). Five outbreak-associated cases died (Table 1).

Collectively, *L. pneumophila* has been the sole causative agent in 9 of 17 outbreaks since 2000. Seven of the outbreaks were attributed to *L. pneumophila* serogroup 1 (Lpsg1), with the source identified as either cooling towers (three outbreaks), spa pools (3) or rainwater tanks (1). Two further outbreaks were attributed to display spa pools caused by Lpsg2.

*L. longbeachae* caused six outbreaks attributed to compost or potting mix exposure, with five caused by *L. longbeachae* serogroup 1 (Llsg1) and one caused by Llsg2. A further outbreak in 2015 attributed to cooling towers caused mixed infections with Lpsg1 and Llsg1 (which were isolated from the towers), along with infections with Lpsg5, Llsg2, and *L sainthelensi* (which weren’t isolated from the towers).

A further cooling tower outbreak was caused by *L. dumoffii*.

Eight of the 17 outbreaks occurred in spring, while four each occurred in summer and autumn, compared to only one during winter. The measured *Legionella* concentration for 10 outbreaks varied widely depending on the identified source, ranging from 47 cfu/mL (public spa pool) to 2400 cfu/mL (cooling tower). Overall, the *Legionella* level in cooling towers involved in outbreaks was 1-to-2 logs higher than those seen in recreational water sources (Table 1).

### Clinical Case Source Investigation Data

Supplementary Table A1 describes individual clinical cases with an epidemiological link to an environmental source for the *Legionella* infection during the 5-year period from 2016 to 2020. A source was classified as definite, probable, or suspect, as described in the methods. A total of 17 different source categories were identified as contaminated with *Legionella* bacteria between 2016 and 2020, with 14 definitive, 67 probable, and 541 suspected sources, giving a total of 622 cases. The most commonly identified sources associated with *Legionella* infections were compost and gardening activities with a total of 571 cases (Supplementary Table A1). Water-based systems only accounted for 40 cases (Supplementary Table A1).

### Environmental Isolate Data

Between 2000 and 2020, a total of 2245 separate environmental isolates of *Legionella* bacteria were identified from a variety of sources, the majority identified at the species level (Supplementary Table A2). Supplementary Table A2 shows the diversity of species that have been isolated from the environment, since 2000 as part of routine source investigation for sporadic and outbreak cases, as well as for risk assessment and surveillance sampling. *Legionella* spp. isolated from water sources such as cooling towers ranked the highest (*N* = 816) followed by compost/mulch/potting mix (*N* = 671) and buildings with complex water distribution systems such as hospitals, aged care homes, and hotels (*N* = 483).

The most common *Legionella* species isolated from hot water distribution systems was *L. anisa* (*N* = 191, or 39.5%), while the most common isolate from cooling towers was *L. pneumophila* (*N* = 544, or 66.7%). *L. longbeachae* isolates made up 55.1% (*N* = 370) of the 671 isolates from compost, with *L. pneumophila* isolates contributing 20.4% (*N* = 137) and *L. bozemanae* being the next most common with 71 isolates (10.6%). Although there was an observed increase in *L. longbeachae* isolates from compost between the two decades of note, is that an increase in other species, such as *L. pneumophila* was also detected in the same organic source categories between the two decades, increasing from *N* = 24, 2000–2009, to *N* = 133, 2010–2020.

Our examination of environmental sources for *Legionella* infection in NZ shows considerable diversity, with both water and soil-based sources contributing to sporadic and outbreak cases. Isolate testing also detected less commonly encountered pathogenic (e.g., *L. gratiana, L. parisiensis,* and *L. steelei*) and non-pathogenic (e.g., *L. moravica* and *L. tunisiensis*) *Legionella* species from a limited number of ecological niches.

The data reviewed here indicate that cooling towers play a key role as the sources of recognized legionellosis outbreaks in NZ. Environmental sampling confirmed the common association of *L. pneumophila* with cooling towers and *L. longbeachae* with compost. However, it also revealed that such associations are not absolute and that some commonly isolated environmental species are not common human pathogens, for example, *L. anisa.* This finding is likely to be due to the difference in comparative pathogenicity of *L. anisa* compared to *L. pneumophila* [11].

This study confirms that recognized outbreaks make only a small contribution to legionellosis case totals in NZ. However, where a source was identified it was always one where aerosolization provides a mechanism for wide dispersion of the contaminated material, be it water or compost. Table 1 records all reported outbreaks since 2000 and shows that although cooling towers were the attributed source of 29.4% (5/17) of outbreaks, they accounted for more than half (58.7 or 54/92) of outbreak-associated legionellosis cases. Spa pools were also attributed as the source of 29.4% (5/17) of outbreaks, but only 14.1% (13/92) of these cases, while compost material was attributed to more than a third (35.3% or 6/17) of all outbreaks, but only 14.1% (13/92) of cases. The significant difference in number of cases associated with each of the major contamination sources is probably due to different dispersal factors and environmental conditions. Cooling towers are common, especially in urban and industrial settings, and capable of discharging aerosolized water contaminated with *Legionella* bacteria over a wide area, potentially exposing many people in the immediate environs as well as those further afield [12]. Sporadic cases of legionellosis are very rarely linked to cooling towers, primarily because these are not routinely sampled when undertaking source investigation for individual legionellosis cases, and this may indicate a deficiency in current testing and sampling protocols. Of note is that there is no widely accepted definition of dangerous *Legionella* concentration levels in the water of cooling tower systems internationally [13].

Spa pools have been linked to both outbreaks and sporadic cases of legionellosis, and their preponderance in such cases reflects their widespread use, both privately and publicly. Invariably, cases are restricted to those that have been exposed to aerosols generated either actively by water jets or by passive dispersion from the water surface and are limited to those in the immediate vicinity of the spa pool. The cases do not have to be users of the pool to be infected. Invariably, investigations for both spa pool and cooling tower outbreaks have identified that the major contributing factors to their cause are inadequate maintenance and cleaning regimes.

Outbreaks and sporadic cases of legionellosis attributed to compost and other soil conditioners follow the inhalation of aerosolized dust containing *Legionella* bacteria [14] and often occur during spring months [3]. Aerosols from this material are usually created when bagged material is emptied or mechanically or manually moved [5]. Invariably, close contact (less than two metres) is required and outbreaks in this setting usually involve two or more people moving large volumes of material or sustained exposure for long periods of time (hours, not minutes). Anecdotally, there have been reports of family members moving a ‘trailer load of compost by shovel and wheelbarrow around the garden’ before presenting days later with symptoms and subsequently being diagnosed with legionellosis. Physical exertion may result in deeper or more rapid breathing, increasing the likelihood of infection following exposure.

In 2012 a *L. pneumophila* sg1 cluster of 19 cases with two deaths occurred in the Greater Auckland areas between March and May of that year [15]. Despite epidemiological, laboratory, and geospatial investigations revealing no common source or sources, it was widely suspected that poorly maintained cooling towers were the likely cause and so mass dosing of all such towers with biocide was recommended via media releases [16]. In the absence of a mandatory register of wet cooling towers, a bylaw was introduced in Auckland requiring owners to register their industrial wet cooling tower systems annually, to monitor *Legionella* bacteria levels, regularly clean and maintain their cooling towers, and send test results to the Council. There have been no recorded outbreaks of legionellosis due to Lpsg1 in the five years since the bylaw was adopted in 2015.

The results of this analysis show that there is considerable potential to improve the completeness of source identification, which would provide a stronger evidence base for action to prevent infections. Supplementary Table A1 shows that several reservoirs were classified as a suspected source as the supporting evidence was weak, usually relying on self-reporting with no environmental sampling. The justification for identification as a suspected source is based on a probabilistic scenario with the elimination of all common sources except the one implicated and is based on information obtained from case interviews. However, these reservoirs and sources may play an important role in the transmission of *Legionella* bacteria, resulting in an underestimation of their importance as a source without physical examination and by attempting to culture legionellae from them. In the absence of regulation aimed at reducing *Legionella* growth and transmission and to optimize source investigation, it is imperative that knowledge about all potential sources of *Legionella* is obtained [17].

A more systematic approach to environmental sampling for *Legionella* could also improve the evidence base to guide prevention measures. Table A2 points to a decrease in active environmental surveillance in contrast to clinical surveillance, which appears to have increased throughout the study period. These findings raise concerns since environmental surveillance is a crucial component of proactive risk assessment and supports actions to reduce exposure to *Legionella* spp. and prevent infections. Any risk assessment for control measures against *Legionella* should be performed not only in response to laboratory-identified cases of disease, but also on a regular basis to prevent the disease [18]. For example, it is noteworthy the gradual decline in NZ since 2014 (2014; *N* = 79, 2020: *N* = 11) of positive isolates from cooling towers being referred to the centralised laboratory for further confirmatory testing and typing which, as evidenced by this study, is of concern since they have been responsible for three outbreaks since 2010 (Table 1). Previous studies have demonstrated that *Legionella* colonization of cooling towers is common, so it would be prudent to assume that all towers have some degree of colonization [19]. It is noteworthy that, as with other jurisdictions [20], clinical cases of Lp have been matched with Lp detected in soil/potting mix/compost samples. This reinforces the importance of considering exposures to organic matter (soils, potting mix, and compost) when investigating cases of LD, especially in circumstances where there is an absence of exposure to an artificial or natural water reservoir.

The main strength of this study is the use of pooled data on *Legionella* environmental reservoirs from laboratory and disease notification data, which highlighted the diversity of *Legionella* species from a variety of environmental sources. Supplementary Tables A1 and A2 list several different artificial or natural reservoirs that support the proliferation and transmission of *Legionella* species to susceptible human hosts most likely due to the aerosolized material containing legionellae. The sources listed in Supplementary Table A2 invariably include water systems that often contain stagnant or recirculating warm water, or reservoirs composed of rich organic material (compost, potting mixes, sewage effluent, and leaf mulch). However, a limitation of this study is associated with the use of routinely collected surveillance data. It is possible that environmental sources of *Legionella* have been detected outside of ESR, as direct notification of laboratory-confirmed environmental *Legionella* samples is not mandatory in NZ.

## Conclusion

The implications of this research are that the environmental ecology of *Legionella* species including species that are infrequently reported, remains pertinent to the prevention of legionellosis. The findings of our study point to the need to put more emphasis on developing control measures in NZ particularly in relation to industrial water systems (cooling towers) and compost exposure. Our findings indicate that *Legionella* can potentially be a significant hazard in high-risk settings such as hospitals and aged and residential care facilities. For example, 71.2%, *N* = 341/479 isolates identified from hot-water systems came from healthcare settings. As a potential public health risk, consideration is needed of how *Legionella* can be managed in such settings and thought should be given to making environmental sources of *Legionella* notifiable in NZ.

A greater understanding of the environmental sources of *Legionella* will also improve targeted prevention efforts. The value of environmental surveillance data would be enhanced if all test requests included a standard set of key data, including the reasons for testing.

## Supplementary Information

Below is the link to the electronic supplementary material.

Supplementary file1 (DOCX 93 KB)

## Data Availability

All data generated or analysed during this study are included in this published article.
