# Supplementary material for: Environmental Investigation and Surveillance for Legionella in Aotearoa New Zealand, 2000–2020
Source: Curr Microbiol. 2023 Mar 30;80(5):156. doi: 10.1007/s00284-023-03261-9 (PMC10063469; doi:10.1007/s00284-023-03261-9)
Supplement: Supplementary file 1 — Supplementary file1 (DOCX 93 KB) [file 284_2023_3261_MOESM1_ESM.docx]

**Supplementary Table A1** Legionellosis cases with their associated exposure source (definitive, probable, suspected, and unknown), by year, 2016–2020

| ***Legionella* source** |  | **Number of clinical cases by level of evidence** | | | | | | | | | | | | | | | | | | | | | | | |
| --- | --- | --- | --- | --- | --- | --- | --- | --- | --- | --- | --- | --- | --- | --- | --- | --- | --- | --- | --- | --- | --- | --- | --- | --- | --- |
|  | **Year** | **2016** | | | | **2017** | | | | **2018** | | | | **2019** | | | | **2020** | | | | **Total**  **2016-2020** | | | |
|  | **Infective agent** | **D** | **P** | **S** | **U** | **D** | **P** | **S** | **U** | **D** | **P** | **S** | **U** | **D** | **P** | **S** | **U** | **D** | **P** | **S** | **U** | **D** | **P** | **S** | **U** |
| **Compost** | *L. longbeachae* (sg unidentified) |  | 14 | 59 |  |  | 6 | 67 |  |  |  | 37 |  |  | 1 | 36 |  |  | 1 | 45 |  |  | 22 | 244 |  |
|  | *L. longbeachae* sg 1 | 7 | 5 | 43 |  |  | 3 | 57 |  |  |  | 29 |  |  | 7 | 30 |  |  | 3 | 32 |  | 7 | 18 | 191 |  |
|  | *L. longbeachae* sg 2 |  |  | 5 |  |  |  | 2 |  |  |  | 1 |  |  | 1 | 1 |  |  |  |  |  |  | 1 | 9 |  |
|  | *L. longbeachae* sg 1 & 2* |  | 1 |  |  |  |  |  |  |  |  |  |  |  |  |  |  |  |  |  |  |  | 1 |  |  |
|  | *L. pneumophila* (sg unidentified) |  |  |  |  |  |  |  |  |  |  | 1 |  |  |  | 2 |  |  | 1 | 4 |  |  | 1 | 7 |  |
|  | *L. pneumophila* sg 1 |  |  |  |  |  |  | 1 |  |  | 1 |  |  |  | 1 | 2 |  |  | 1 | 1 |  |  | 3 | 4 |  |
|  | *L. pneumophila* sg 5 |  |  |  |  |  |  | 1 |  |  |  |  |  |  |  |  |  |  |  | 1 |  |  |  | 2 |  |
|  | *L. bozemanae/L. longbeachae* |  |  |  |  |  |  | 2 |  |  |  |  |  |  |  |  |  |  |  |  |  |  |  | 2 |  |
|  | *L. bozemanae/L. jordanis* |  |  |  |  |  |  |  |  |  |  |  |  |  |  |  |  |  |  | 2 |  |  |  | 2 |  |
|  | *L. bozemanae* sg 1 |  |  |  |  |  |  | 1 |  |  |  | 2 |  |  |  | 2 |  |  |  |  |  |  |  | 5 |  |
|  | *L. bozemanae* sg 2 |  |  | 2 |  |  |  |  |  |  |  |  |  |  |  |  |  |  |  |  |  |  |  | 2 |  |
|  | *L. dumoffii/L. longbeachae* |  |  |  |  |  |  |  |  |  |  | 1 |  |  |  |  |  |  |  |  |  |  |  | 1 |  |
|  | *L. feeleii* sg 1 |  |  |  |  |  |  |  |  |  |  | 1 |  |  |  |  |  |  |  |  |  |  |  | 1 |  |
|  | *L. hackeliae* |  |  |  |  |  |  |  |  |  |  |  |  |  |  | 1 |  |  |  |  |  |  |  | 1 |  |
|  | *L. jordanis* |  |  | 1 |  |  |  |  |  |  | 1 |  |  |  |  |  |  |  |  |  |  |  | 1 | 1 |  |
|  | *L. micdadei* |  |  | 1 |  |  |  |  |  |  |  | 3 |  |  |  |  |  |  |  | 2 |  |  |  | 6 |  |
|  | *L. micdadei/L. longbeachae* |  |  | 1 |  |  |  |  |  |  |  |  |  |  |  |  |  |  |  |  |  |  |  | 1 |  |
|  | *L. sainthelensi* |  |  | 2 |  |  |  | 3 |  |  |  | 1 |  |  |  | 1 |  |  |  |  |  |  |  | 7 |  |
|  | *Legionella* spp. (unidentified) |  | 1 | 3 |  |  |  | 2 |  |  |  | 7 |  |  |  |  |  |  |  | 1 |  |  | 1 | 13 |  |
| **Gardening activities** | *L. longbeachae* (sg unidentified) |  |  | 2 |  |  |  |  |  |  |  |  |  |  |  | 1 |  |  |  | 1 |  |  |  | 5 |  |
|  | *L. longbeachae* sg 1 |  |  | 3 |  |  |  |  |  |  |  | 1 |  |  |  | 1 |  |  |  | 2 |  |  |  | 7 |  |
|  | *L. dumoffii* |  |  |  |  |  |  |  |  |  |  |  |  |  |  | 1 |  |  |  |  |  |  |  | 1 |  |
|  | *L. micdadei* |  |  |  |  |  |  |  |  |  |  |  |  |  |  | 1 |  |  |  |  |  |  |  | 1 |  |
|  | *L. pneumophila* sg 1 |  |  |  |  |  |  |  |  |  |  | 1 |  |  |  |  |  |  |  | 1 |  |  |  | 2 |  |
|  | *L. pneumophila* sg 2 |  |  |  |  |  |  |  |  |  |  |  |  |  |  | 1 |  |  |  |  |  |  |  | 1 |  |
| **Mulch** | *L. longbeachae* (sg unidentified) |  |  | 1 |  |  |  |  |  |  |  |  |  |  |  |  |  |  |  |  |  |  |  | 1 |  |
| **Soil (incl. excavation/ construction; excl. gardening or compost use)** | *L. longbeachae* (sg unidentified) |  |  | 2 |  |  |  | 1 |  |  |  |  |  |  |  | 1 |  |  |  | 1 |  |  |  | 5 |  |
|  | *L. longbeachae* sg 1 |  |  | 1 |  |  |  | 1 |  |  |  | 1 |  |  |  |  |  |  |  |  |  |  |  | 3 |  |
|  | *L. dumoffii* |  |  |  |  |  |  | 1 |  |  |  |  |  |  |  |  |  |  |  |  |  |  |  | 1 |  |
| **Woodchip** | *L. bozemanae* sg 1 |  |  |  |  |  |  |  |  |  |  |  |  |  |  |  |  |  |  | 1 |  |  |  | 1 |  |
| **Nosocomial** | *L. pneumophila* sg 1 | 1 |  |  |  | 1 |  |  |  |  | 1 |  |  |  |  |  |  |  |  |  |  | 2 | 1 |  |  |
| **Cooling tower** | *L. jordanis* |  |  |  |  |  |  |  |  |  |  | 1 |  |  |  |  |  |  |  |  |  |  |  | 1 |  |
| **Drinking water (vessel)** | *L. pneumophila* sg 10 |  |  |  |  | 1 |  |  |  |  |  |  |  |  |  |  |  |  |  |  |  | 1 |  |  |  |
| **Drinking water (rainwater tank)** | *L. pneumophila* sg 1 |  |  |  |  |  |  |  |  | 1 |  |  |  |  |  |  |  |  | 1 |  |  | 1 | 1 |  |  |
| **Drinking water (groundwater bore)** | *L. pneumophila* sg 1 |  |  |  |  |  |  |  |  |  | 1 |  |  |  |  |  |  |  |  |  |  |  | 1 |  |  |
|  | *L. pneumophila* (sg unidentified) |  |  |  |  |  |  |  |  |  |  |  |  |  |  |  |  |  |  | 1 |  |  |  | 1 |  |
| **Domestic/hotel hot water system** | *L. pneumophila* sg 1 | 1 |  |  |  |  |  |  |  |  |  |  |  |  | 1 |  |  |  |  |  |  | 1 | 1 |  |  |
|  | *L. pneumophila* (sg unidentified) |  |  |  |  |  |  |  |  |  | 1 |  |  |  |  |  |  |  |  |  |  |  | 1 |  |  |
| **Drainage system** | *L. pneumophila* sg 12 |  |  | 1 |  |  |  |  |  |  |  |  |  |  |  |  |  |  |  |  |  |  |  | 1 |  |
| **Roof guttering/clearing** | *L. sainthelensi* |  |  |  |  |  |  | 1 |  |  |  |  |  |  |  |  |  |  |  |  |  |  |  | 1 |  |
| **Geothermal pool/water** | *L. pneumophila* sg 1 |  |  |  |  |  |  |  |  |  |  |  |  |  | 1 |  |  |  |  |  |  |  | 1 |  |  |
|  | *L. sainthelensi* |  |  |  |  |  |  |  |  |  |  | 1 |  |  |  |  |  |  |  |  |  |  |  | 1 |  |
| **Spa pool** | *L. pneumophila* (sg unidentified) |  |  | 1 |  |  |  |  |  |  |  |  |  |  | 2 |  |  |  |  |  |  |  | 2 | 1 |  |
|  | *L. pneumophila* sg 1 |  | 2 | 2 |  |  |  |  |  |  | 2 |  |  |  |  | 1 |  |  | 4 |  |  |  | 8 | 3 |  |
|  | *L. pneumophila* sg 2 | 1 |  |  |  |  |  |  |  |  |  |  |  |  |  |  |  |  |  |  |  | 1 |  |  |  |
|  | *L. pneumophila* sg 6 |  |  |  |  |  |  |  |  |  |  |  |  |  |  |  |  |  | 1 |  |  |  | 1 |  |  |
|  | *L. pneumophila* sg 13 |  |  |  |  |  |  |  |  |  |  |  |  |  |  |  |  |  | 1 |  |  |  | 1 |  |  |
|  | *L. pneumophila* (other e.g 91-033) |  |  |  |  | 1 |  |  |  |  |  |  |  |  |  |  |  |  |  |  |  | 1 |  |  |  |
|  | *L. bozemanae* sg 2 |  |  | 1 |  |  |  |  |  |  |  |  |  |  |  |  |  |  |  |  |  |  |  | 1 |  |
| **Swimming pool** | *L. pneumophila* sg 1 |  |  |  |  |  |  | 1 |  |  |  |  |  |  |  |  |  |  |  |  |  |  |  | 1 |  |
| **Water blaster** | *L. pneumophila* sg 1 |  |  |  |  |  |  |  |  |  |  | 1 |  |  |  | 1 |  |  |  |  |  |  |  | 2 |  |
| **CPAP machine** | *L. pneumophila* sg 1 |  |  |  |  |  | 1 |  |  |  |  |  |  |  |  |  |  |  |  |  |  |  | 1 |  |  |
|  | *L. pneumophila* sg 15 |  |  | 1 |  |  |  |  |  |  |  |  |  |  |  |  |  |  |  |  |  |  |  | 1 |  |
| **Total (known source)** | | **10** | **23** | **132** |  | **3** | **10** | **141** |  | **1** | **7** | **89** |  | **0** | **14** | **83** |  | **0** | **13** | **96** |  | **14** | **67** | **541** |  |
| **Foreign travel (no sampling)** | *L. dumoffii* |  |  |  | 1 |  |  |  |  |  |  |  | 1 |  |  |  |  |  |  |  |  |  |  |  | 2 |
|  | *L. longbeachae* (sg unidentified) |  |  |  | 1 |  |  |  |  |  |  |  |  |  |  |  |  |  |  |  |  |  |  |  | 1 |
|  | *L. longbeachae* sg 1 |  |  |  | 1 |  |  |  |  |  |  |  | 1 |  |  |  |  |  |  |  | 1 |  |  |  | 3 |
|  | *L. micdadei* |  |  |  |  |  |  |  |  |  |  |  |  |  |  |  | 1 |  |  |  |  |  |  |  | 1 |
|  | *L. pneumophila* (sg unidentified) |  |  |  |  |  |  |  | 1 |  |  |  | 2 |  |  |  | 2 |  |  |  |  |  |  |  | 5 |
|  | *L. pneumophila* sg 1 |  |  |  | 6 |  |  |  | 3 |  |  |  | 4 |  |  |  | 3 |  |  |  | 1 |  |  |  | 17 |
|  | *L. pneumophila* sg 2 |  |  |  |  |  |  |  |  |  |  |  | 1 |  |  |  |  |  |  |  |  |  |  |  | 1 |
|  | *L. pneumophila* sg 4 |  |  |  | 1 |  |  |  |  |  |  |  |  |  |  |  | 1 |  |  |  |  |  |  |  | 2 |
|  | *L. pneumophila* sg 12 |  |  |  | 2 |  |  |  | 1 |  |  |  |  |  |  |  |  |  |  |  |  |  |  |  | 3 |
| **Total (Foreign travel -no sampling)** | |  |  |  | **12** |  |  |  | **5** |  |  |  | **9** |  |  |  | **7** |  |  |  | **2** |  |  |  | **35** |
| **Cases where no source identified (no environmental sampling undertaken)**  **Cases where no source (no environmental sampling undertaken)** | *L. bozemanae* sg 1 |  |  |  | 1 |  |  |  |  |  |  |  |  |  |  |  |  |  |  |  |  |  |  |  | 1 |
|  | *L. bozemanae* sg 2 |  |  |  |  |  |  |  | 1 |  |  |  |  |  |  |  |  |  |  |  |  |  |  |  | 1 |
|  | *L. dumoffii* |  |  |  | 1 |  |  |  | 2 |  |  |  | 2 |  |  |  | 3 |  |  |  |  |  |  |  | 10 |
|  | *L. dumoffii/L longbeachae* |  |  |  |  |  |  |  | 2 |  |  |  |  |  |  |  |  |  |  |  |  |  |  |  | 2 |
|  | *L. gormanii* |  |  |  |  |  |  |  | 1 |  |  |  |  |  |  |  |  |  |  |  |  |  |  |  | 1 |
|  | *L. hackeliae* |  |  |  |  |  |  |  |  |  |  |  | 2 |  |  |  |  |  |  |  |  |  |  |  | 2 |
|  | *L. jordanis* |  |  |  |  |  |  |  |  |  |  |  |  |  |  |  |  |  |  |  | 1 |  |  |  | 1 |
|  | *L. longbeachae* (sg unidentified) |  |  |  | 7 |  |  |  | 4 |  |  |  | 8 |  |  |  | 7 |  |  |  | 12 |  |  |  | 38 |
|  | *L. longbeachae* sg 1 |  |  |  | 11 |  |  |  | 6 |  |  |  | 10 |  |  |  | 8 |  |  |  | 5 |  |  |  | 40 |
|  | *L. longbeachae* sg 2 |  |  |  | 5 |  |  |  | 1 |  |  |  | 1 |  |  |  | 3 |  |  |  |  |  |  |  | 10 |
|  | *L. micdadei* |  |  |  | 4 |  |  |  | 1 |  |  |  | 2 |  |  |  |  |  |  |  |  |  |  |  | 7 |
|  | *L. oakridgensis* |  |  |  | 2 |  |  |  |  |  |  |  |  |  |  |  |  |  |  |  |  |  |  |  | 2 |
|  | *L. pneumophila* (other e.g 97-2898) |  |  |  |  |  |  |  |  |  |  |  | 1 |  |  |  |  |  |  |  |  |  |  |  | 1 |
|  | *L. pneumophila* (sg unidentified) |  |  |  | 5 |  |  |  | 6 |  |  |  | 1 |  |  |  | 8 |  |  |  | 5 |  |  |  | 25 |
|  | *L. pneumophila* sg 1 |  |  |  | 17 |  |  |  | 27 |  |  |  | 37 |  |  |  | 22 |  |  |  | 21 |  |  |  | 124 |
|  | *L. pneumophila* sg 1or6 |  |  |  |  |  |  |  | 1 |  |  |  |  |  |  |  |  |  |  |  |  |  |  |  | 1 |
|  | *L. pneumophila* sg 1or12 |  |  |  | 1 |  |  |  |  |  |  |  |  |  |  |  |  |  |  |  |  |  |  |  | 1 |
|  | *L. pneumophila* sg 2 |  |  |  | 2 |  |  |  | 1 |  |  |  | 1 |  |  |  | 1 |  |  |  |  |  |  |  | 5 |
|  | *L. pneumophila* sg 4 |  |  |  |  |  |  |  | 1 |  |  |  | 1 |  |  |  |  |  |  |  |  |  |  |  | 2 |
|  | *L. pneumophila* sg 6 |  |  |  | 1 |  |  |  |  |  |  |  |  |  |  |  |  |  |  |  |  |  |  |  | 1 |
|  | *L. pneumophila* sg 7 |  |  |  |  |  |  |  | 1 |  |  |  | 1 |  |  |  | 1 |  |  |  |  |  |  |  | 3 |
|  | *L. pneumophila* sg 8 |  |  |  |  |  |  |  | 1 |  |  |  |  |  |  |  |  |  |  |  |  |  |  |  | 1 |
|  | *L. pneumophila* sg 12 |  |  |  | 5 |  |  |  | 2 |  |  |  | 1 |  |  |  |  |  |  |  |  |  |  |  | 8 |
|  | *L. pneumophila* sg 13 |  |  |  |  |  |  |  | 1 |  |  |  | 1 |  |  |  |  |  |  |  |  |  |  |  | 2 |
|  | *L. pneumophila* sg 14 |  |  |  |  |  |  |  |  |  |  |  |  |  |  |  |  |  |  |  | 1 |  |  |  | 1 |
|  | *L. sainthelensi* |  |  |  | 3 |  |  |  | 1 |  |  |  | 2 |  |  |  | 1 |  |  |  | 3 |  |  |  | 10 |
|  | *L. wadsworthii* |  |  |  |  |  |  |  | 1 |  |  |  |  |  |  |  |  |  |  |  |  |  |  |  | 1 |
|  | *Legionella* spp. (unidentified) |  |  |  | 4 |  |  |  | 1 |  |  |  | 4 |  |  |  | 2 |  |  |  | 2 |  |  |  | 13 |
|  | *Legionella* D3582 |  |  |  | 2 |  |  |  |  |  |  |  |  |  |  |  | 1 |  |  |  |  |  |  |  | 3 |
| **Total unknown** |  |  |  |  | **81** |  |  |  | **67** |  |  |  | **84** |  |  |  | **64** |  |  |  | **52** |  |  |  | **348** |
| **Total of all cases** |  | **248** | | | | **221** | | | | **182** | | | | **161** | | | | **161** | | | | **973** | | | |
| Abbreviations: Definitive – D; Probable – P; Suspected – S; Unknown – U; CPAP machine: Continuous Positive Airway Pressure Machine. * = Dual infection | | | | | | | | | | | | | | | | | | | | | | | | | |
| Note: blank cells indicate a case count of zero (0) | | | | | | | | | | | | | | | | | | | | | | | | | |

**Supplementary Table A2** *Legionella* culture isolates by infective organism (species and serogroup) and environmental source category, total for 2000–2020

| **Infective organisms** | **Compost/ mulch/ potting mix/ soil-** | | **Cooling towers** | | **Hot water distribution systems** | | **Cold water distribution system** | | **Industrial process water** | | **Recreational water (incl. geothermal)** | | **Effluent/sewage** | | **Miscellaneous#** | | **Total all sources** | |
| --- | --- | --- | --- | --- | --- | --- | --- | --- | --- | --- | --- | --- | --- | --- | --- | --- | --- | --- |
|  | **No.** | **Proportion%** | **No.** | **Proportion%** | **No.** | **Proportion%** | **No.** | **Proportion%** | **No.** | **Proportion%** | **No.** | **Proportion%** | **No.** | **Proportion%** | **No.** | **Proportion%** | **No.** | **Proportion**  **%** |
| **Total** | **671** | **100** | **816** | **100** | **483** | **100** | **35** | **100** | **84** | **100** | **131** | **100** | **16** | **100** | **9** | **100** | **2245** | **100** |
| *L. anisa* | 7 | 1.0 | 92 | 11.3 | 191 | 39.5 | 1 | 2.9 | 8 | 9.5 | - | - | - | - | - | - | 299 | 13.3 |
| *L. birminghamensis* | 3 | 0.4 | - | - | 3 | 0.6 | - | - | - | - | - | - | - | - | - | - | 6 | 0.3 |
| *L. bozemanae* sg? | 9 | 1.3 | - | - | 3 | 0.6 | - | - | - | - | - | - | - | - | - | - | 12 | 0.5 |
| *L. bozemanae* sg1 | 45 | 6.7 | 8 | 1.0 | 3 | 0.6 | - | - | 1 | 1.2 | - | - | - | - | - | - | 57 | 2.5 |
| *L. bozemanae* sg2 | 17 | 2.5 | 2 | 0.2 | - | - | - | - | - | - | - | - | - | - | - | - | 19 | 0.8 |
| *L. cherrii* | - | - | 2 | 0.2 | - | - | - | - | - | - | - | - | - | - | - | - | 2 | 0.1 |
| *L. cincinnatiensis* | 2 | 0.3 | - | - | - | - | - | - | - | - | - | - | - | - | - | - | 2 | 0.1 |
| *L. donaldsonii* | - | - | - | - | - | - | - | - | - | - | 1 | 0.8 | - | - | - | - | 1 | 0.04 |
| *L. dumoffii* | 4 | 0.6 | 2 | 0.2 | 4 | 0.8 | - | - | - | - | 2 | 1.5 | - | - | 1 | 11.1 | 13 | 0.6 |
| *L. erythra* | - | - | 1 | 0.1 | 1 | 0.2 | - | - | 2 | 2.4 |  |  | - | - | - | - | 4 | 0.2 |
| *L. feeleii* sg? | 3 | 0.4 | 5 | 0.6 | - | - | - | - | - | - | - | - | 3 | 18.8 | 1 | 11.1 | 13 | 0.6 |
| *L. feeleii* sg1 | 2 | 0.3 | 18 | 2.2 | 1 | 0.2 | 5 | 14.3 | 1 | 1.2 | - | - | 1 | 6.3 | 2 | 22.2 | 29 | 1.3 |
| *L. feeleii* sg2 | 2 | 0.3 | 9 | 1.1 | 2 | 0.4 | - | - | 1 | 1.2 | - | - | - | - | - | - | 14 | 0.6 |
| *L. geestiana* | - | - | - | - | 14 | 2.9 | - | - | - | - | - | - | - | - | 1 | 11.1 | 15 | 0.7 |
| *L. gormanii* | 12 | 1.8 | - | - | - | - | - | - | - | - | - | - | 1 | 6.3 | - | - | 13 | 0.6 |
| *L. gratiana* | 2 | 0.3 | - | - | - | - | - | - | - | - | - | - | 1 | 6.3 | - | - | 3 | 0.1 |
| *L. hackeliae* | - | - | 1 | 0.1 | - | - | - | - | - | - | - | - | - | - | - | - | 1 | 0.04 |
| *L. jamestowniensis* | 2 | 0.3 | 1 | 0.1 | 1 | 0.2 | - | - | - | - | - | - | - | - | - | - | 4 | 0.2 |
| *L. jordanis* | 4 | 0.6 | - | - | - | - | - | - | 1 | 1.2 | 2 | 1.5 | - | - | - | - | 7 | 0.3 |
| *L. londiniensis* | - | - | - | - | 16 | 3.3 | - | - | - | - | - | - | 2 | 12.5 | - | - | 18 | 0.8 |
| ***L. longbeachae total*** | **370** | **55.1** | **4** | **-** | **-** | **-** | **1** | **2.9** | **-** | **-** | **1** | **0.8** | **1** | **6.3** | **-** | **-** | **377** | **16.8** |
| *L. longbeachae* sg? | 7 | 1.0 | - | - | - | - | - | - | - | - | - | - | 1 | 6.3 | - | - | 8 | 0.4 |
| *L. longbeachae* sg1 | 309 | 46.1 | 3 | 0.4 | - | - | 1 | 2.9 | - | - | 1 | 0.8 | - | - | - | - | 314 | 14 |
| *L. longbeachae* sg2 | 54 | 8.0 | 1 | 0.1 | - | - | - | - | - | - | - | - | - | - | - | - | 55 | 2.5 |
| *L. maceachernii* | - | - | 1 | 0.1 | - | - | - | - | 3 | 3.6 | - | - | - | - | - | - | 4 | 0.2 |
| *L. micdadei* | 18 | 2.7 | 2 | 0.2 | 1 | 0.2 | - | - | 1 | 1.2 | 11 | 8.4 | - | - | - | - | 33 | 1.5 |
| *L. moravica* | 1 | 0.1 | - | - | - | - | - | - | - | - | - | - | - | - | - | - | 1 | 0.04 |
| *L. nautarum* | - | - | - | - | - | - | - | - | - | - | 2 | 1.5 | - | - | - | - | 2 | 0.1 |
| *L. oakridgensis* | - | - | 1 | 0.1 | - | - | - | - | - | - | - | - | - | - | - | - | 1 | 0.04 |
| *L. parisiensis* | 3 | 0.4 | - | - | - | - | - | - | - | - | - | - | - | - | - | - | 3 | 0.1 |
| ***L. pneumophila total*** | **137** | **20.4** | **544** | **66.7** | **221** | **45.8** | **26** | **74.3** | **59** | **70.2** | **102** | **77.9** | **-** | **-** | **3** | **33.3** | **1100** | **49.0** |
| *L. pneumophila* sg? | 18 | 2.7 | 17 | 2.1 | 2 | 0.4 | 1 | 2.9 | 3 | 3.6 | 1 | 0.8 | 3 | 18.8 | - | - | 45 | 2 |
| *L. pneumophila* sg1 | 32 | 4.8 | 227 | 27.8 | 150 | 31.1 | 7 | 20.0 | 38 | 45.2 | 59 | 42.0 | - | - | 3 | 33.3 | 516 | 23.0 |
| *L. pneumophila* sg10 | 26 | 3.9 | 26 | 3.2 | 1 | 0.2 | 5 | 14.3 | - | - | - | - | - | - | - | - | 58 | 2.6 |
| *L. pneumophila* sg12 | 3 | 0.4 | - | - | - | - | - | - | - | - | - | - | - | - | - | - | 3 | 0.1 |
| *L. pneumophila* sg13 | 5 | 0.7 | 7 | 0.9 | 1 | 0.2 | 2 | 5.7 | 2 | 2.4 | - | - | - | - | - | - | 17 | 0.8 |
| *L. pneumophila* sg14 | 2 | 0.3 | 4 | 0.5 | - | - | - | - | 2 | 2.4 | - | - | - | - | - | - | 8 | 0.4 |
| *L. pneumophila* sg2 | - | - | 5 | 0.6 | 2 | 0.4 | - | - | - | - | 9 | 6.9 | - | - | - | - | 16 | 0.7 |
| *L. pneumophila* sg3 | 13 | 1.9 | 26 | 3.2 | - | - | - | - | 2 | 2.4 | 4 | 3.1 | - | - | - | - | 45 | 2 |
| *L. pneumophila* sg4 | 7 | 1.0 | 22 | 2.7 | 2 | 0.4 | - | - | - | - | 3 | 2.3 | - | - | - | - | 34 | 1.5 |
| *L. pneumophila* sg5 | 1 | 0.1 | 49 | 6.0 | 8 | 1.7 | - | - | 2 | 2.4 | 6 | 4.6 | - | - | - | - | 66 | 2.9 |
| *L. pneumophila* sg6 | 12 | 1.8 | 110 | 13.5 | 29 | 6.0 | 7 | 20.0 | 5 | 6.0 | 10 | 7.6 | - | - | - | - | 173 | 7.7 |
| *L. pneumophila* sg7 | 3 | 0.4 | 3 | 0.4 | 4 | 0.8 | - | - | - | - | 3 | 2.3 | 2 | 12.5 | - | - | 15 | 0.7 |
| *L. pneumophila* sg8 | 2 | 0.3 | 45 | 5.5 | 15 | 3.1 | - | - | 5 | 6.0 | 6 | 4.6 | - | - | - | - | 73 | 3.3 |
| *L. pneumophila* sg9 | - | - | - | - | - | - | - | - | - | - | 1 | 0.8 | - | - | - | - | 1 | 0.04 |
| *L. pneumophila* strain 91-033 | 13 | 1.9 | 2 | 0.2 | - | - | - | - | - | - | - | - | - | - | - | - | 15 | 0.7 |
| *L. pneumophila* strain 97-2898 | - | - | 1 | 0.1 | 7 | 1.4 | 4 | 11.4 | - | - | 3 | 2.3 | - | - | - | - | 15 | 0.7 |
| *L. quateirensis* | - | - | - | - | 5 | 1.0 | - | - | - | - | - | - | - | - | - | - | 5 | 0.2 |
| *L. quinlivanii* | 1 | 0.1 | 18 | 2.2 | - | - | - | - | 1 | 1.2 | - | - | - | - | - | - | 20 | 0.9 |
| *L. rubrilucens* | - | - | 47 | 5.6 | 1 | 0.2 | 2 | 5.7 | 2 | 2.4 | - | - | - | - | - | - | 52 | 2.3 |
| *L. sainthelensi* sg? | 3 | 0.4 | 5 | 0.6 | - | - | - | - | - | - | - | - | 1 | 6.3 | - | - | 9 | 0.4 |
| *L. sainthelensi* sg1 | - | - | 2 | 0.2 | - | - | - | - | - | - | - | - | - | - | - | - | 2 | 0.1 |
| *L. sainthelensi* sg2 | 3 | 0.4 | 2 | 0.2 | - | - | - | - | - | - | - | - | - | - | - | - | 5 | 0.2 |
| *L. santicrucis* | 2 | 0.3 | 3 | 0.4 | - | - | - | - | - | - | - | - | - | - | - | - | 5 | 0.2 |
| *L. spiritensis* | - | - | 1 | 0.1 | - | - | - | - | - | - | - | - | - | - | - | - | 1 | 0.04 |
| *L. steelei* | - | - | - | - | 1 | 0.2 | - | - | - | - | - | - | - | - | - | - | 1 | 0.04 |
| L. species L-29 | - | - | 2 | 0.2 | 1 | 0.2 | - | - | 1 | 1.2 | - | - | - | - | - | - | 4 | 0.2 |
| L. species 7322 | - | - | - | - | - | - | - | - | 1 | 1.2 | - | - | - | - | - | - | 1 | 0.04 |
| L. species D5382 | 2 | 0.3 | - | - | - | - | - | - | - | - | - | - | - | - | - | - | 2 | 0.1 |
| L. species H | - | - | - | - | - | - | - | - | - | - | 1 | 0.8 | - | - | - | - | 1 | 0.04 |
| L. strain IMVS-97L11 | - | - | - | - | 1 | 0.2 | - | - | - | - | - | - | - | - | - | - | 1 | 0.04 |
| *L. taurinensis* | - | - | 4 | 0.5 | 1 | 0.2 | - | - | - | - | - | - | - | - | - | - | 5 | 0.2 |
| *L. tunisiensis* | - | - | - | - | - | - | - | - | - | - | 1 | 0.8 | - | - | - | - | 1 | 0.04 |
| *L. wadsworthii* | - | - | - | - | 2 | 0.4 | - | - | - | - | - | - | 1 | 6.3 | - | - | 3 | 0.1 |
| *L. waltersii* | - | - | - | - | 1 | 0.2 | - | - | - | - | - | - | - | - | - | - | 1 | 0.04 |
| L. species unidentified | 17 | 2.5 | 38 | 4.7 | 9 | 1.9 | - | - | 3 | 3.6 | 5 | 3.8 | - | - | 1 | 11.1 | 73 | 3.6 |

# irrigation systems, medical/respiratory equipment, ornamental water features
